# Postoperative intussusception in infants and children: a report of seven cases^☆^

**DOI:** 10.1016/S1674-8301(12)60009-8

**Published:** 2012-01

**Authors:** Weiwei Jiang, Weibing Tang, Qiming Geng, Xiaoqun Xu

**Affiliations:** Department of Neonatal Surgery, Nanjing Children's Hospital Affiliated with Nanjing Medical University, Nanjing, Jiangsu 210029, China.

**Keywords:** postoperative intussusception, intestinal obstruction, infants, children

## Abstract

Postoperative intussusception is an uncommon but serious condition in infants and children. Here, we report seven cases of postoperative intussusception in infants and children who were seen at our institution over the last 13 y. The patients showed increased nasogastric drainage, vomiting, lack of stool, and/or growing abdominal distension 2 to 9 d following abdominal surgery. Manual reduction was successful in five cases. In two cases, necrosis was found and intestinal resection and anastomosis were carried out. No recurrence was observed at six months of follow-up. Postoperative intussusception should be suspected in pediatric surgical patients who showed signs of intestinal obstruction in the early postoperative period.

## INTRODUCTION

Though intestinal obstruction is common in infants and children after abdominal surgery, postoperative intussusception is rarely seen in these young patients[1]. Intussusception is the invagination of one segment of the bowel into another, causing bowel obstruction. Postoperative intussusception occurs as a complication of many surgical procedures, including appendicectomy and surgery for intestinal malrotation, Meckel's diverticulum, retroperitoneal teratoma, Wilm's tumor, and Hirschsprung' disease[2]-[5].

A delayed or incorrect diagnosis of acute intussusception can have serious consequences. Physicians often do not suspect the presence of acute intussusception if the condition occurs early in the postoperative period when patients often have atypical clinical manifestations, rendering a prompt and correct diagnosis difficult. Patients with intussusception frequently do not exhibit the classical triad of abdominal pain, sausage-shaped palpable mass and bloody stools, which can be misdiagnosed as intestinal adhesions.

Here, we report the successful management of seven cases of acute postoperative intussusception who sought treatment at our institution in the past 13 y.

## CASE REPORT

**Fig. 1:**
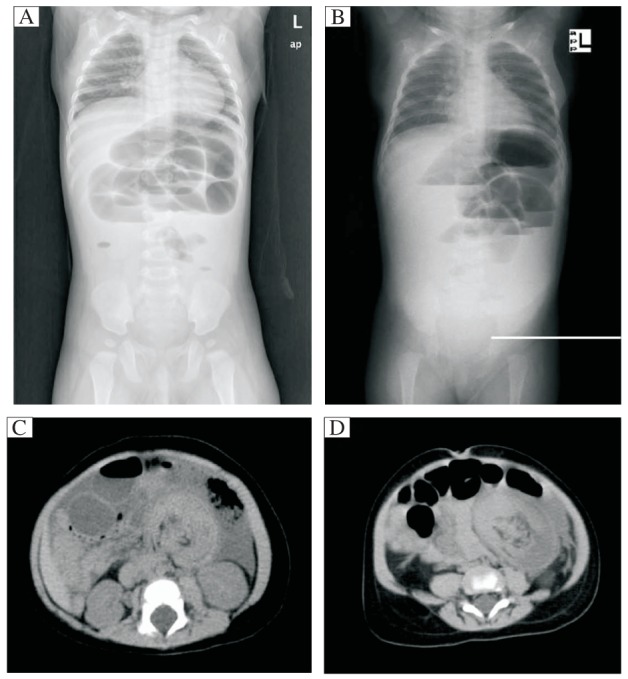
The imaging examination of patients. A and B: Abdominal X-ray showed there was an intestinal obstruction in children. C and D: CT showed that there was an sign of concentric circle with intestinal intussusception preoperatively.

Between January 1997 and December 2009, 7 children including 5 boys and 2 girls, whose age ranged from 4 mon to 5 y with a median age of 12.2 mon, were admitted to our hospital because of symptoms of intestinal obstruction, which developed following abdominal surgery. These children had sought surgical treatment due to teratoma, Hirschsprung' disease, ileocolic intussusception, appendicitis, incarcerated inguinal hernia, or congenital choledochal cyst (***Table 1***). Two to nine d after the surgical procedures, they experienced increased nasogastric drainage, vomiting, lack of stool and/or growing abdominal distension. Abdominal X-ray examinations showed the presence of intestinal obstruction in these children (***Fig. 1A*** and ***Fig. 1B***). CT showed the presence of concentric circle preoperatively (***Fig. 1C*** and ***Fig. 1D***).

**Table 1:** Primary disease and original operations for 7 children

Primary disease	Cases	Age	Gendre	Location	Original operations
Teratoma	1	5mon	male	ileum	Abdominal teratoma resection
Hirschsprung' disease	1	6mon	female	ileum	Radical macrosigmoid operation
	1	5mon	male	ileum	Ileocolic intussusception manual reduction
Ileocolic intussusception	1	5mon	male	ileum	Ileocolic intussusception intestines resection and anastomosis
Appendicitis	1	5y	male	ileum	Appendicectomy
Incarcerated inguinal hernia	1	1 y and 2 mon	male	ileum	Incarcerated inguinal hernia reduction
Congenital choledochal cyst	1	4mon	female	ileum	Kasai operation

The latter finding was atypical. Intestinal obstruction was diagnosed and intussusception was suspected.

**Fig. 2:**
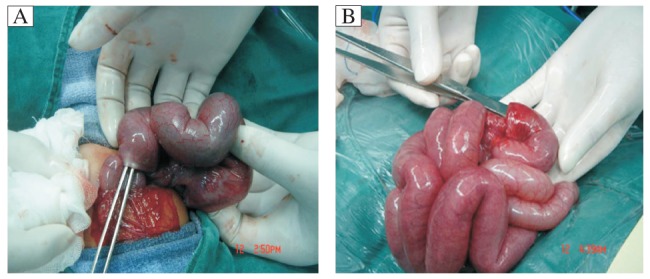
Intraoperative photographs showing that the intussusception was located at the ileum.

Laparotomy was performed in these patients. In 5 patients, manual reduction was successful and intestinal necrosis was not examined, while in the other two, intestinal necrosis was confirmed and intestine resection and anastomosis were performed. The intussusception was located at the ileum and was 5-30 cm long (***Fig. 2***). There was no recurrence during six mon of follow-up.

## DISCUSSION

Postoperative intussusception is a complication of abdominal surgery in children, and it is difficult to predict preoperatively. The incidence of this complication is about 0.05%, in our patients and the age of these patients fell between 4 mon and 5 y with a median of 12.2 mon. Patients with postoperative intussusception may experience increased nasogastric drainage, vomiting, lack of stool and growing abdominal distension, and they rarely exhibit the classical triad of abdominal pain, sausage-shaped palpable mass and bloody stools[6],[7]. Our patients developed symptoms of intestinal obstruction due to intestinal intussusception usually within 10 postoperative days. Patients with obstruction due to adhesion may not show symptoms of bowel obstruction until more than 2 w after surgery, which can help clinicians to distinguish the two causes of intussusception. Postoperative intussusception should be suspected if the intestinal obstruction occurs in less than 10 postoperative days.

Many theories have been put forward to explain the etiology of postoperative intussusception, but the underlying mechanism is unclear. Common causes of intussusception include appendicectomy, and surgery for malrotation, Meckel's diverticulum, retroperitoneal teratoma, Wilm's tumor and Hirschsprung' disease[2]-[5]. In particular, it may result from surgical trauma, spasm, tissue dehydration, electrolyte imbalance, hypoxia and/or edema[8]-[10]. An earlier study demonstrated that lipopolysaccharide could induce postoperative intussusception as intussusception occurred in mice injected intraperitoneally with lipopolysaccharide[11],[12].

The majority of postoperative obstructions result from intestinal adhesions, but the possibility of intestinal intussusception should not be overlooked. Clinical signs and symptoms of postoperative intussusception are nonspecific; therefore, abdominal plain films and laboratory data are of little diagnostic value in these cases. However, ultrasound and computed tomography are capable of delineating the features of intestinal intussusception[13]. Early laparotomy is necessary when enema reduction is unsuccessful. Clinicians should be aware of this uncommon complication in children in the early postoperative period.
